# Emotional Eating Beyond Obesity: A Transdiagnostic Conceptual Analysis of Phenotyping, Measurement, and Priorities for Clinical Validation

**DOI:** 10.3390/nu18203399

**Published:** 2026-10-16

**Authors:** Octavian Vasiliu, Andrei G. Mangalagiu, Diana Gabriela Vasiliu, Bogdan M. Petrescu

**Affiliations:** 1 Discipline of Psychiatry II, Neurosciences Department, Faculty of Medicine, “Carol Davila” University of Medicine and Pharmacy, 020021 Bucharest, Romania; octavian.vasiliu@umfcd.ro (O.V.); bogdan.petrescu@umfcd.ro (B.M.P.); 2 Clinical Department of Psychiatry, Central Military Emergency University Hospital “Dr. Carol Davila”, 010825 Bucharest, Romania; 3 Department of Internal Medicine, Mallow General Hospital, P51 N288 Mallow, Cork, Ireland; dianaojog@gmail.com

**Keywords:** emotional eating, eating disorders, diet quality, eating behavior, obesity, emotion dysregulation, transdiagnostic, ecological momentary assessment, clinical validation

## Abstract

*Background*: The concept of emotional eating (EE) originates in the early psychosomatic accounts of overeating, but its nosological status remains contested more than four decades after it was first scientifically explored. Although neither the DSM-5-TR nor the ICD-11 recognizes EE as a discrete entity, clinically significant presentations of EE are described in the literature and can currently be captured under residual diagnostic categories in the absence of specific criteria. *Objective*: To examine whether EE is best conceptualized as an independent disorder or a dimensional, transdiagnostic, nutritional-behavioral phenotype, and to identify requirements for its clinical validation. *Methods*: A conceptual analysis supported by a critical synthesis of the literature focused on phenotypic heterogeneity, nutritional correlates, measurement approaches, diagnostic boundaries, and clinical implications of EE. *Results*: Negative-affect EE (N-EE) has been linked to changes in food choice, diet quality, processing degree, eating patterns, and potential abdominal adiposity, but these associations do not resolve the contentious question of whether EE is a distinct clinical entity. Eating in response to emotional states has also been linked to depression, emotion dysregulation, dietary restraint, binge eating, body-image concerns, sleep disturbance, and obesity-related outcomes. However, these associations are heterogeneous and do not define a unique disorder. One important asymmetry further complicates classification: N-EE is more consistently associated with psychopathology, while positive EE (P-EE) often occurs in normative social contexts. The proposed framework positions EE as a dimensional, transdiagnostic phenotype and identifies convergent multimethod validity, clinically meaningful thresholds, and discriminant, longitudinal, nutritional, prognostic, and treatment validity beyond established eating and mood disorders as requirements for potential categorical recognition. *Conclusions*: The literature considered suggests that EE may be usefully approached as a dimensional, transdiagnostic nutritional-behavioral phenotype. This remains a provisional conceptual position rather than an empirically validated classification. Further multimethod and longitudinal research is needed to clarify clinical significance, diagnostic boundaries, and utility before considering independent diagnostic recognition.

## 1. Introduction: From a Common Behavior to a Candidate Clinical Phenotype

In this Perspective, emotional eating (EE) refers broadly to changes in eating behavior associated with emotional states, including increases or decreases in intake, changes in food choice, meal timing or eating patterns, and perceived control over eating [1,2,3,4]. Many studies operationalize EE more narrowly as eating more in response to negative emotions; their findings should therefore be interpreted according to the specific definition and measure used. Emotional influences may coexist with physiological hunger, and neither loss of control nor clinically significant distress is required for the broad descriptive construct. The origins of the concept under discussion can be traced back more than four decades, but both early and contemporary accounts have emphasized more or less the same aspects: negative affect, hyperpalatable food, coping, and learned associations between distress and eating [1,5,6,7,8,9]. The construct is clinically appealing because it provides a plausible bridge between affect regulation and eating behavior, and meta-analytic research has reported associations between difficulties in emotional regulation and manifestations of eating disorders (ED) in adolescents and young adults of both sexes [10]. EE, loss-of-control eating, and disordered eating were associated with emotion-regulation difficulties in subgroup analyses [10], supporting their clinical co-occurrence without establishing that emotional dysfunction causes these eating behaviors. However, the broad categorical scope of EE creates a classification problem, i.e., rather than representing a discrete disorder, this may be referring to a coping style, a vulnerability factor, a symptom dimension, or a manifestation embedded within another eating, mood, or metabolic condition [1,6,7,8,11].

The precursors of EE are concepts from early psychosomatic accounts of obesity, which have been based on the observation that some individuals learn to respond to emotional arousal by eating because internal emotional states are misinterpreted as hunger [12,13]. Subsequent research has shifted the focus toward deliberate dietary restriction and the possibility that disruption of cognitive control could produce disinhibited or counterregulatory eating, particularly under emotional or situational pressure [14]. The development of instruments such as the Dutch Eating Behavior Questionnaire (DEBQ) and the Emotional Eating Scale (EES) subsequently transformed EE from a largely theoretical explanation into a measurable construct, further supporting its conceptual validity [15,16,17]. More recent research has expanded the definition of EE by distinguishing positive from negative emotional eating and by comparing retrospective questionnaire scores with ecological and laboratory measures [5,14,15,16]. This evolution has increased the clinical visibility of EE while also broadening its conceptual boundaries, thus contributing to the current uncertainty about whether it represents a stress-related phenomenological manifestation, an emotion-regulation pattern (either conscious or unconscious), a trait marker, or a potentially independent clinical phenotype [2,3,5,14,15,16,18,19,20].

Several influential models are useful for explaining why the emergence of EE does not imply that a unique diagnosis can be assigned. For example, the psychosomatic and emotion-regulation view conceptualizes eating as a strategy for modulating aversive internal states; restraint-based accounts emphasize the vulnerability created by dieting and disinhibition; conditioning models propose that repeated pairing of negative affect and food intake can make eating an acquired response; and contemporary integrative models emphasize interactions among mood, sleep, interoception, reward, self-regulation, body image, and environmental context [5,8,11,14,18]. From the broader perspective of nutritional psychiatry, the relationships between eating patterns and mental health are similarly understood to be multifactorial, potentially involving inflammatory, metabolic, microbiome-related, and neurobehavioral pathways, although the therapeutic evidence remains heterogeneous and limited by substantial methodological variability [21]. The mechanisms previously listed are not mutually exclusive, and when considered together, their coexistence helps explain why the same EE score may arise from different causal pathways.

The strongest argument for the clinical relevance of EE comes from converging associations with patient-relevant outcomes. Prospective studies have shown that depressive symptoms have been associated with higher levels of EE, which, in turn, predicts later increases in BMI and waist circumference, particularly among individuals with shorter sleep durations [22]. In adolescence, poorer emotional regulation predicted later EE, whereas weight status and negative body image modified this association [23]. These longitudinal associations provide information about temporal ordering but do not, by themselves, establish causation or eliminate confounding by sleep, mood symptoms, dietary restraint, or other relevant factors [22,23]. Associations between EE and younger age, female sex, self-regulation difficulties, fast-food consumption, and unhealthy dietary patterns have also been presented in population studies [24,25], whereas stress, anxiety, boredom, guilt, and perceived loss of control have been highlighted in qualitative research as lived experiences of such behavior [26]. These findings support the importance of EE not only for mental health but also for physical equilibrium, although none of them, in isolation, can be considered sufficient to establish categorical diagnostic independence.

Previous reviews have examined EE mechanisms, its relationship with obesity, multimethod assessment, and treatment, while meta-analyses have quantified selected associations and intervention outcomes [1,4,11,27,28,29,30,31]. The contribution proposed here is focused on integration of these findings around a specific question: what evidence would distinguish a clinically useful EE phenotype from an independent disorder? The framework separates emotional valence, behavioral expression, and clinical impact, and links these dimensions to questions about measurement, diagnostic boundaries, temporality, nutrition, prognosis, and treatment utility. Overall, this framework is an organizing proposal for research, not a new diagnostic system or a claim that EE’s transdiagnostic nature has not previously been recognized.

The main objective of the current analysis is to explore whether EE should currently be regarded as a clinically meaningful transdiagnostic phenotype rather than a discrete psychiatric disorder, and to identify the empirical conditions under which categorical recognition could eventually become justified.

## 2. Methods

This conceptual analysis draws on a focused critical synthesis of foundational and contemporary literature, emphasizing developments published in the last ten years. Sources were identified through searches of PubMed/MEDLINE, Web of Science, and Google Scholar up to 15 September 2026, supplemented by examination of reference lists. Search terms addressed emotional eating, emotion regulation, dietary restraint, nutritional correlates, assessment, and diagnostic validity. Theoretical accounts, observational and experimental studies, psychometric investigations, evidence syntheses, and intervention studies were considered for their relevance to five analytical domains: phenotypic heterogeneity, nutritional correlates, measurement, diagnostic boundaries, and clinical implications. The search process was iterative, and the exact database-specific search strings used across successive searches were not retained, limiting the reproducibility of the search. Search terms were refined as relevant concepts and publications emerged.

Foundational publications informed the conceptual background, while empirical studies and evidence syntheses informed the discussion of these domains, including inconsistent or conflicting findings. The synthesis distinguished evidence of association and treatment responsiveness from evidence supporting diagnostic independence and informed the proposed dimensional framework and validation priorities. The approach was intended to develop a testable conceptual position rather than provide exhaustive coverage or a formal assessment of evidence certainty.

## 3. Emotional Eating Is Heterogeneous in Regard to Mechanism, Valence, and Context

### 3.1. Emotional Regulation, Restraint, Conditioning, and Body-Image Pathways

Emotional dysregulation is among the most robust correlates of EE, and a large body of research supports this assertion. A 2026 meta-analysis of 40 studies and 42 samples (*N* = 14,481) revealed a moderate positive association between broad emotion dysregulation and EE overall (pooled r = 0.34, 95% CI [0.28, 0.40]); the association remained significant when the study was restricted to negative emotional eating (pooled r = 0.29, 95% CI [0.23, 0.35]) but was nonsignificant for positive emotional eating (pooled r = 0.06, 95% CI [−0.11, 0.22]) [28]. These contemporary findings are consistent with earlier longitudinal and clinical observations in which reduced emotional control, depressive affect, and difficulty identifying or managing internal states are linked to EE [5,11,23,32]. However, emotional dysregulation is itself transdiagnostic and is prominent across the ED spectrum [32]; therefore, it can explain the clinical salience of EE without providing evidence that this phenomenon constitutes in itself a separate disorder. Also, it is important to note that these pooled correlations do not establish that emotion dysregulation causes EE.

Restrained eating offers a second avenue for analyzing the pathogenesis of EE. Classic work on dietary restraint has proposed that self-imposed rigid restrictions can generate deprivation, disinhibition, and episodes of overeating when an individual’s cognitive control is challenged [14,18]. Emotional eating may overlap with this process, but the constructs are not synonymous: cluster-analytic work in normal-weight women has revealed that only certain restrained eaters were in fact emotional eaters and that only certain emotional eaters displayed bulimic tendencies [7]. More recent meta-analytic evidence has likewise indicated a weaker association between EE and restraint than between EE and binge eating (d = 0.20 vs. d = 0.53; both *p* < 0.001) [29]. This type of finding suggests that restraint eating can amplify EE expression in a subgroup without universally defining the phenotype.

**Figure 1:**
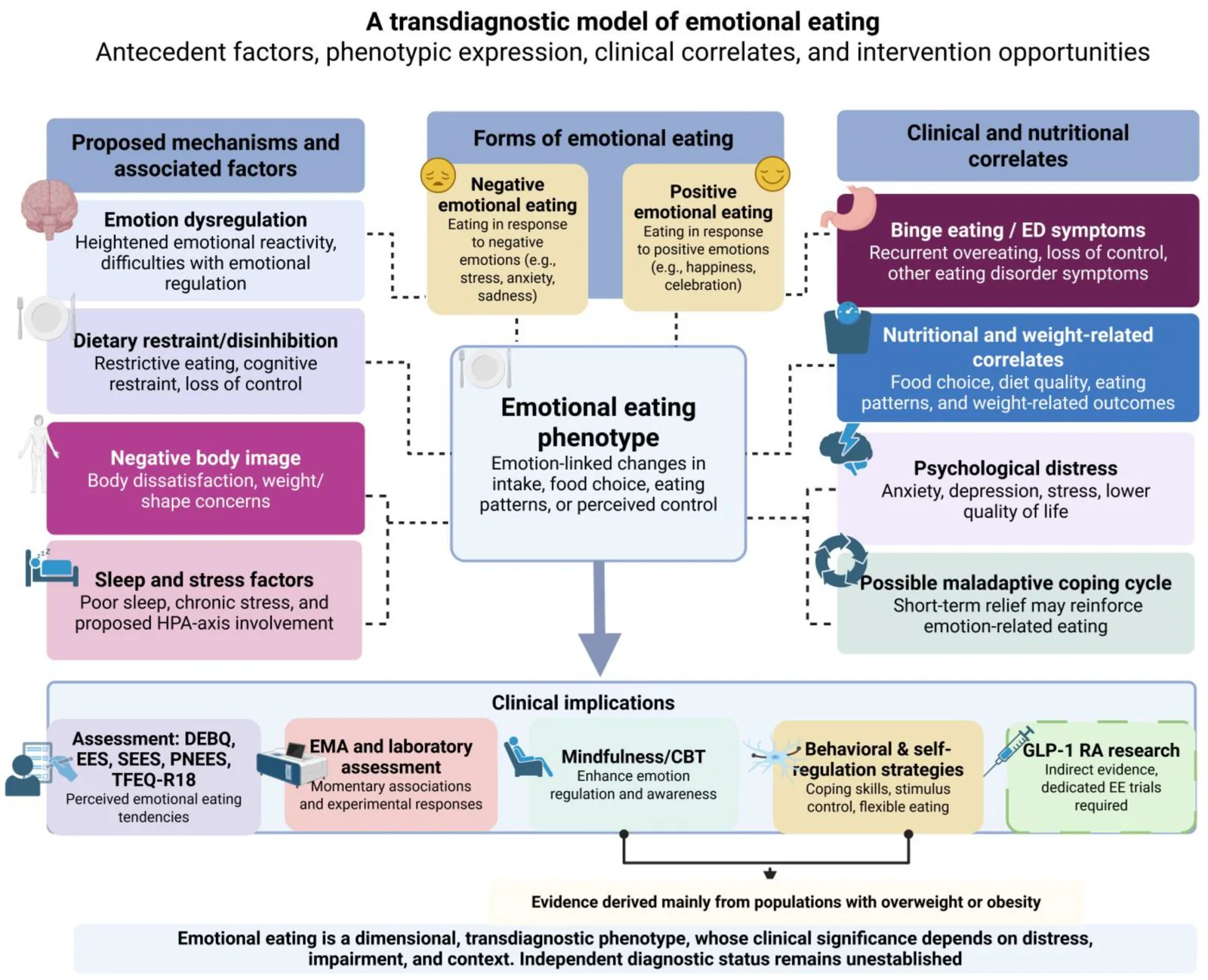
**A transdiagnostic model of emotional eating: antecedent mechanisms, phenotypic expression, clinical correlates, and intervention opportunities.** The diagram represents a conceptual framework rather than a validated causal model, and the direction of the arrows should not be interpreted as indicating causality. The GLP-1 RA panel reflects indirect evidence only, and dedicated EE trials are required. Abbreviations: CBT, cognitive behavioral therapy; DEBQ, Dutch Eating Behavior Questionnaire; ED, eating disorder; EES, Emotional Eating Scale; EMA, ecological momentary assessment; GLP-1 RA, glucagon-like peptide-1 receptor agonist; HPA, hypothalamic–pituitary–adrenal; PNEES, Positive–Negative Emotional Eating Scale; SEES, Salzburg Emotional Eating Scale; and TFEQ-R18, 18-item Three-Factor Eating Questionnaire. Created in BioRender. Vasiliu, O. (2026). https://BioRender.com/nr4pq2x.

Conditioning and reward processes provide a third possible explanation. EE has been associated with the severity of binge-eating among overweight individuals [8], and the repeated pairing of unpleasant affect with food intake may strengthen learned expectancies that suggest that eating will relieve distress [1]. This premise echoes the escape-from-self-awareness model of binge eating, which similarly proposes that eating functions to narrow attention away from an aversive, self-critical state [33], and is complemented by the reward-based stress eating model, which links cortisol, HPA-axis activity, and mesolimbic reward circuitry to the preferential intake of calorically dense food under stress [34], motivating the HPA-axis hypothesis depicted in Figure 1 without establishing causal mediation in EE. However, this pathway may interact with body image and self-efficacy, and weight loss studies have shown that mood, eating-related self-efficacy, and self-control can change in conjunction with EE [35]. The result of exploring these directions of research is best understood as a network of mutually reinforcing mechanisms rather than a single pathognomonic mechanism, since each has arguments in its favor yet lacks the power to epistemologically exhaust the topic.

### 3.2. Positive and Negative Emotional Eating Should Not Be Conflated

A major conceptual advance in this area has been the recognition that eating in response to positive emotions, the so-called P-EE, is not simply the mirror image of N-EE. The Positive–Negative Emotional Eating Scale (PNEES) has been used to demonstrate that positive and negative EE can have different associations with overeating and binge eating [2]. The Salzburg Emotional Eating Scale (SEES) separates sadness-, anger-, anxiety-, and happiness-related eating and accounts for both increases and decreases in intake in response to emotions [3]. More recently, a 2025 scoping review of positive EE among college students revealed that positive EE does not map consistently onto psychopathology and may coexist with adaptive or socially normative eating patterns; the fact that P-EE may be a significant correlate of healthy eating habits among college students raises the question of the necessary limits when exploring the EE phenomenon, in order to avoid its overpathologization [36].

Continuing this direction of analysis, the distinction between positive and negative emotional eating carries direct nosological implications. A construct cannot be considered pathological merely because it is influenced by emotion, and since eating is embedded in reward, culture, celebration, social bonding, and daily routines, care should be taken when exploring the border between normality and pathology. The clinically relevant question is then whether the emotional trigger is associated with recurrent loss of control, distress, functional impairment, and persistence in the face of adverse consequences or whether it represents a pattern that meaningfully predicts other psychiatric or metabolic outcomes. N-EE is more likely to satisfy some of these conditions, but it remains heterogeneous, whereas P-EE seems to often occur in normative contexts. At any rate, emotional valence by itself cannot determine clinical significance, and both P-EE and N-EE need to be evaluated in relation to distress, functional impact, and degree of control over eating.

The proposed relationships among antecedent vulnerabilities, negative and positive emotional eating, clinical correlates, assessment approaches, and treatment opportunities are summarized in Figure 1. In this framework, (1) EE arises from interactions among psychological, behavioral, nutritional, and contextual factors, including emotion dysregulation, dietary restraint/disinhibition, negative body image, and sleep- and stress-related influences; (2) the clinical correlates may include binge eating and other eating-disorder symptoms, changes in food choice or diet quality, weight-related outcomes, psychological distress, and the reinforcement of maladaptive coping cycles; (3) assessment should combine validated self-report instruments with ecological and experimental approaches and, when relevant, dietary phenotyping; and (4) interventions, in turn, may target emotion regulation, cognitive and behavioral mechanisms, self-regulation, nutritional behavior, and biological pathways; the pharmacological evidence remains indirect in relation to an EE-specific indication.

## 4. Nutritional and Clinical Distribution: Common, Cross-Diagnostic, and Not Reducible to Obesity

The occurrence of EE has been described in various community samples comprising children, adolescents, young adults, individuals with metabolic disorders, and patients with EDs. In a U.S. national study (*N* = 5863 participants), 20.5% of adults reported emotionally driven overeating (often or very often), with sociodemographic (i.e., female gender, non-Hispanic White, and younger age) and self-regulation correlates (i.e., incapacity to delay gratification) [24]. In a Saudi sample of healthy young women (*N* = 638 participants, ages 18–39) assessed during the COVID-19 pandemic, 12.4% had high-severity EE scores, and these scores correlated negatively with increased family income and positively with fat intake, number of meals, sugar consumption, BMI, stress, energy intake, and fast food intake frequency [37]. In a cross-sectional study among Hong Kong undergraduates (*N* = 424 participants, aged 18–24 years), women reported negative EE more frequently than men did; mild depressive symptoms were associated independently with N-EE in males, comparatively with females, where the most consistent associations were not having a romantic partner, having depressive symptoms, and at least mild stress [38]. In a 12-country, 5-continent pediatric sample (N = 5426 participants, aged 9–11 years), EE was associated with unhealthy dietary patterns across cultural settings and in both genders, suggesting EE is in no way a limited phenomenon to Western countries or to a specific gender [25]. Eating behaviors during adolescence are also influenced by cognitive and motivational determinants that extend beyond emotional triggers. In a study of female adolescents (N = 400 participants, mean age 14.9+/−0.78 years), attitudes and perceived behavioral control jointly explained 34% of the variance in healthy-eating intentions, which illustrates the importance of distinguishing emotional drivers from the broader determinants of dietary behavior [39]. These results show that prevalence is strongly dependent on the population, the instrument used for assessment, and the threshold for detecting EE.

Obesity increases the clinical visibility of EE but should not be included in its definition, as available data support its consideration only as a co-occurrence. A 2025 meta-analysis of 18 studies (N = 21,237) estimated a pooled prevalence of 44.9% among individuals with overweight or obesity, with substantial heterogeneity (*I*^2^ = 98.7%), which was partly attributable to differences in measurement and not to differences in geographical distribution of the individuals [40]. A 2026 meta-analysis of 41 studies also revealed higher EE scores in individuals with elevated BMIs, although the effect was modest (SMD = 0.27, 95% CI [0.18, 0.36]). The association was moderated by developmental stage, remaining nonsignificant in young individuals (SMD = −0.04) while occurring in adults [41]. Similarly, earlier work has emphasized the notion that EE can occur in people without obesity and that weight status alone does not explain emotion-eating patterns, with subsequent research only adding evidence to support this observation [5,6].

From a nutritional perspective, the phenotype should not be reduced to total caloric intake, specific food preferences, or body mass. A recent synthesis of negative-affect EE has suggested that changes in food choice, diet quality, degree of food processing, and eating pattern may be more consistently associated with EE than changes in intake quantity [4]. Questionnaire-based findings associate self-reported stress with preferences for highly palatable, energy-dense foods, including among some individuals reporting reduced overall intake [42]. Plausible links between EE and abdominal adiposity have also been discussed [27], but the evidence summarized here does not establish a uniform causal pathway or show that visceral fat accumulation is a necessary consequence of EE. These reported associations may be clinically relevant, but should be treated as phenotypic dimensions rather than as defining criteria. Nutritional assessment should therefore be conducted in conjunction with BMI and waist circumference measurements to characterize dietary pattern and quality when these outcomes are relevant.

When approaching established EDs, the evidence is compatible with a phenotypical classification. EE scores are elevated particularly in binge-related phenotypes, but the patterns differ across the well-defined EDs, i.e., bulimia nervosa, binge-eating disorder, and anorexia nervosa subtypes [43]. A 2024 ecological momentary assessment study similarly revealed diagnosis-specific associations between emotional states and food craving across the ED spectrum: binge-eating and food restriction during N-EE in patients with bulimia nervosa and anorexia nervosa, and normalized appetitive responses during P-EE in patients with binge-eating disorder and anorexia nervosa [44]. These data are compatible with a transdiagnostic dimension that cuts across existing categories, without establishing the existence of a syndrome that is clearly separate from them.

## 5. Structured Measurement of EE Is Part of the Nosological Problem

### 5.1. What Questionnaires Measure

Studies into EE have a mature questionnaire tradition, and multiple instruments have already been presented in the previous chapters. The Three-Factor Eating Questionnaire (TFEQ) distinguishes among cognitive restraint, disinhibition, and hunger and can be used to classify eating-style patterns in large populations; the revised 18-item Three-Factor Eating Questionnaire (TFEQ-R18) assesses cognitive restraint, uncontrolled eating, and emotional eating [19,20]. The Dutch Eating Behavior Questionnaire (DEBQ) is used to assess emotional, external, and restrained eating [15]. The Emotional Eating Scale (EES) is focused on eating in response to anger/frustration, anxiety, and depression [16], whereas in the SEES and PNEES, the construct is expanded to specific positive and negative emotions [2,3]. All these tools (and the list is not exhaustive) make EE measurable, reproducible, and clinically approachable from an evidence-based perspective.

However, such diversity does not come without associated problems, since different instruments do not necessarily measure the same phenomenon. Some of them are used to assess perceived tendency towards EE, others are used to assess the frequency or direction of intake, while others are used to assess the relationship with specific emotions. This heterogeneity in outcomes can create seemingly contradictory findings when a slightly different construct is measured in each study. Therefore, a high questionnaire score can reflect a stable belief about one’s eating style, recurrent episodes, sensitivity to emotion, or general distress, making the results of research highly dependent on the alignment among the study’s hypothesis, primary and secondary outcomes, and the tools used for measurement. A categorical diagnosis would require clarification of which dimensions are central to the EE construct and how subjective beliefs relate to momentary eating patterns.

### 5.2. EMAs and Laboratory Studies Reveal Incomplete Convergence

Naturalistic and multimethod research highlights this limitation, as reflected in a 2025 mega-analysis of eight ecological momentary assessment (EMA) datasets (*N* = 764 participants), which found bidirectional associations between affect and food craving and showed that trait eating-style measures mainly moderate concurrent rather than prospective relationships [45]. Another 2025 study (*N* = 56 adults) comparing the EES, a laboratory paradigm, and 14 days of EMA found only partial convergence across methods; emotional eating evaluated on the EES in response to depression, anxiety/anger, and boredom was associated with EMA-measured EE, but individuals with high scores on EES and in daily life did not consume more calories in the lab following a negative mood induction [46]. The study included 56 adults with overweight or obesity, 93% of whom were women, limiting generalization to other weight groups and more sex-balanced populations [46]. In 2026, experience-sampling research provided stronger evidence for time-pressure-driven snacking than for a straightforward momentary emotion-to-snacking pathway in a group of 64 participants, suggesting that changes in snacking behavior are more closely linked to specific situational constraints, e.g., time pressure, than to emotional fluctuations [47]. This incomplete convergence extends to the presumed affect-relief mechanism itself: a meta-analysis of 36 EMA studies of binge eating (*N* = 968 participants) failed to support the affect regulation model, finding that negative affect did not reliably decrease after eating [48], and a meta-analysis of 56 experimental mood-induction studies (*N* = 3670 participants) found that negative emotions increased food intake in restrained eaters but not in individuals with obesity, eating disorders, or self-identified emotional eaters, while positive emotions increased eating across all groups [49].

Retrospective EE questionnaires primarily assess perceived tendencies, recalled experiences, and beliefs about eating in response to emotion. EMA examines momentary affect and eating in daily life and, with suitable temporal sampling, can estimate whether affect precedes reported eating within individuals. These approaches address related but distinct constructs, so incomplete agreement does not by itself make either method invalid [45,46,50]. EMA may also involve self-reported intake, missed episodes, and sampling effects, and temporal precedence alone does not establish causation. Multimethod validation should therefore test convergence where theoretically expected and explain informative differences between subjective attribution and momentary behavioral patterns.

## 6. Is Emotional Eating a Disorder? The Pros and Cons of Nosological Construction

Current diagnostic systems do not recognize EE as a discrete entity, as neither the DSM-5-TR nor the ICD-11 lists this condition among the defined feeding and eating disorders [51,52]. Instead, a clinically significant presentation could currently be captured by using residual categories which do not provide specific criteria for EE, such as Other Specified Feeding or Eating Disorder (OSFED) or Unspecified Feeding or Eating Disorder (UFED) in the DSM-5-TR, and the analogous OSFED (6B8Y) or Feeding or Eating Disorders, Unspecified (6B8Z) in the ICD-11 [51,52]. Notably, within these residual categories, both systems explicitly name night eating syndrome as an example presentation, while emotional eating, despite a large and rapidly growing empirical literature, has no comparably designated pathway. These categories were designed to flag clinically significant presentations that fall outside the defined criteria rather than to operationalize a new construct, as they do not provide thresholds for frequency, severity, distress, or duration, and their use cannot, therefore, resolve the question of whether EE constitutes a bounded clinical entity. This classificatory silence in the currently validated nosographical systems can only motivate, and not substitute for, the evidentiary analysis and interpretative framework provided below.

### 6.1. Arguments Supporting Clinical Recognition

Several features justify systematic clinical assessment of EE. First, it is measurable with multiple validated instruments [2,3,15,16,19,20]; second, EE is associated with distress, binge eating, depression, emotion dysregulation, and weight-related outcomes [22,28,29,40,41,43]; and third, it can identify treatment-relevant difficulties even when BMI is not the primary concern. Behavioral weight loss research suggests that EE warrants specific attention rather than being viewed merely as a byproduct of obesity [53]. The EE construct aligns with dimensional approaches to ED psychopathology, where clinically meaningful traits cross categorical boundaries [54,55,56].

The fourth aspect is its actionability, meaning that if identifying EE helps clinicians select emotion-regulation, cognitive, mindfulness-based, or self-regulation interventions, then the construct has clinical utility regardless of its final nosological status. This aspect is especially relevant because body image and related appearance concerns can intersect with eating behavior in ways that weight alone cannot describe enough [57].

### 6.2. Arguments Against an Independent Diagnosis at Present

The evidence discussed in this narrative analysis does not yet provide sufficient support for recognizing EE as an independent diagnostic category. There are multiple, unresolved questions concerning clinically meaningful thresholds, discriminant validity, measurement consistency, and the distinction between normative and clinically significant emotion-related eating, all of which may shape the future definition of EE. First, there are no generally accepted thresholds for frequency, duration, severity, loss of control, distress, or impairment. Second, the discriminant validity is limited: EE overlaps with binge eating, bulimia nervosa, dietary restraint, depression, anxiety, and obesity-related distress [7,8,28,29,32,43]. Third, measurement depends on the method [45,46,47]. Fourth, positive emotional eating may be normative [2,3,36]. Also, improvement through treatment does not prove diagnostic independence because the interventions that reduce EE can also modify broader processes that are initially difficult to circumscribe. Table 1 summarizes the arguments for and against independent diagnostic recognition of EE, highlighting the advantages of considering such a diagnosis and potential directions for future research needed to evaluate its validity.

Differential diagnosis is especially important when eating occurs at night or in altered states of awareness, making the distinction between binge-eating disorder, night eating syndrome, EE, and other conditions more difficult. For example, sleep-related eating disorders are distinct sleep-related conditions characterized by involuntary or partially conscious eating episodes, and they require a different diagnostic frame [58]. Similarly, binge-eating disorder is characterized by recurrent episodes of objectively large food intake and loss of control over eating, whereas EE can occur without objective binge size [59]. Clinically, EE must also be distinguished from restrained eating, externally cued eating, and appetite changes, which are symptoms of mood disorders, because these constructs may coexist while reflecting distinct proximal mechanisms [60,61]. These boundary problems argue for dimensional assessment prior to categorical expansion, and the differential positioning of EE in relation to these and other clinically overlapping constructs is synthesized in Table 2.

These limitations support further evaluation of EE using dimensional assessment while leaving its potential categorical status open to future empirical investigation.

## 7. Proposed Multidimensional Framework for Emotion-Related Eating and Clinical Significance

We propose a multidimensional framework in which emotional valence, frequency and persistence, behavioral flexibility and perceived control, distress and functional impairment, and nutritional manifestations are assessed separately. These dimensions may vary independently and may change over time, without defining an established sequence of stages. Positive or negative emotional triggers and weight status do not, by themselves, establish clinical significance. The framework is heuristic and hypothesis-generating without being labeled as a validated clinical staging or diagnostic tool and provides no established cutoffs. Whether any combination of dimensions identifies a distinct disorder remains a separate empirical question requiring validation, and may be a topic for further research in this field.

**Figure 2:**
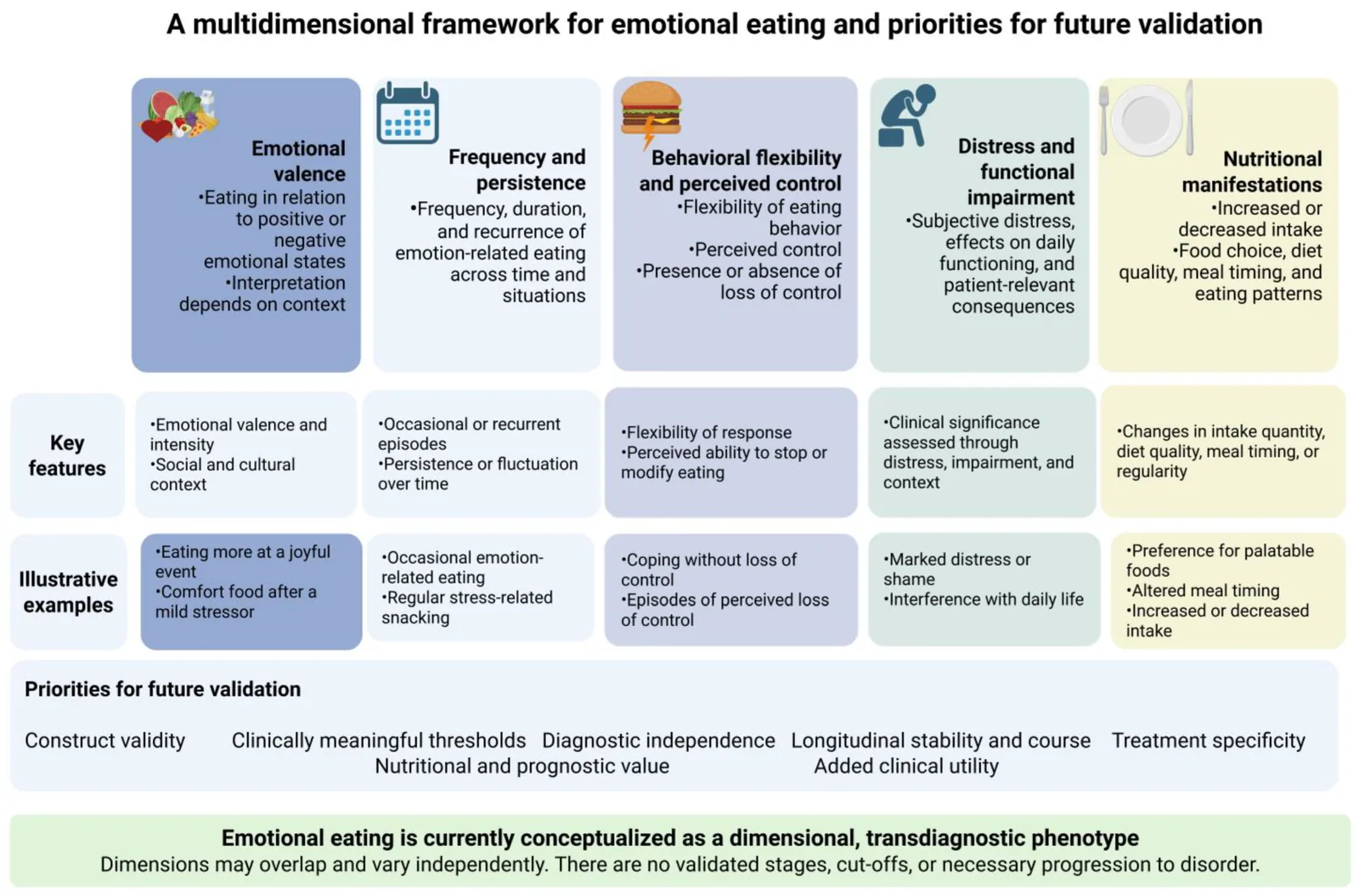
**A multidimensional framework for emotional eating and priorities for future validation.** The domains describe potentially overlapping aspects of emotion-related eating, not successive stages or a necessary trajectory toward disorder. Clinical significance requires assessment of distress, impairment, and context. Candidate validation requirements are presented separately from the phenotypic dimensions; no diagnostic category, threshold, or staging system is established by this figure. Created in BioRender. Vasiliu, O. (2026). https://biorender.com/7739tq2.

The phenotypic dimensions and the separate validation requirements for possible diagnostic recognition are summarized in Figure 2. Diagnostic recognition would require reproducible evidence of construct validity, clinically meaningful thresholds, diagnostic independence from existing disorders, longitudinal stability, nutritional and prognostic value, treatment specificity, and clinical utility. The added value of this framework is intended to lie in connecting descriptive dimensions with testable validation questions, rather than in proposing new stages, validated thresholds, or a single causal mechanism.

Future nosographical definitions should rely on more than a high score from a single scale, regardless of the psychometric properties of such an instrument. The key issue considered here is whether identifying a reproducible phenotype provides diagnostic, prognostic, and therapeutic benefits beyond those of established disorders and helps recognize clinically significant distress or impairment. Table 3 outlines the proposed validation priorities for evaluating emotional eating as a candidate diagnostic construct.

## 8. Nutritional and Treatment Implications: A Target Can Be Clinically Useful Without Being Classified as a Disorder

Treatment research supports the practical value of assessing clinically significant EE within integrated clinical management. Earlier work has suggested that combining exercise with cognitive–behavioral strategies, with changes mediated by self-regulation, self-efficacy, and mood, has potential benefits [66]. Mindfulness-based emotional-eating awareness training and broader mindfulness interventions have also been shown to be beneficial for EE and binge-related symptoms, although the effects on weight are variable [67,68]. These findings are consistent with the idea that the maintenance process, i.e., emotion regulation, automaticity, restraint, or self-efficacy, may be more therapeutically important than the diagnostic label.

**Table 3:** **Proposed validation priorities for evaluating emotional eating as a candidate diagnostic construct.**

Proposed Validation Priorities	Evidence Considered Important	Preferred Approach	Grounded in
***Validity domains***			
**Construct validity**	Convergence where theoretically expected, with informative differences between perceived tendencies and momentary behavioral patterns	Questionnaires, repeated EMA, and laboratory or dietary measures where feasible; analyze subjective attribution and temporal associations separately	[50,69,70]
**Diagnostic boundaries**	Incremental validity beyond BED, BN, mood symptoms, restraint, external eating, BMI, and sleep-related eating	Clinical comparison groups and multivariable models	[69,70]
**Temporal validity**	Distinguish stable between-person tendencies from within-person fluctuations; examine persistence, recurrence, and transitions between reproducible profiles without assuming stages	Large, diverse cohorts with extended follow-up and repeated EMA periods; validate candidate boundaries against outcomes in independent samples	[69,70]
**Prognostic validity**	Prediction of eating-disorder, metabolic, or functional outcomes beyond mood and anxiety symptoms, binge eating, restraint, sleep, and BMI	Longitudinal cohorts with adjustment for established predictors and assessment across weight groups and demographic and cultural settings	[69,70]
***Utility domains***			
**Clinical significance**	Patient-relevant distress, functional impairment, and meaningful clinical benefit beyond changes in EE scores	Assess patient-relevant distress and functional impairment alongside dietary quality and eating patterns; interpret scale change in relation to meaningful clinical benefit.	[69,71]
**Nutritional validity**	Reproducible associations with diet quality, food choice, eating pattern, central adiposity, or metabolic outcomes beyond BMI alone	Dietary assessment combined with repeated eating data and anthropometric/metabolic follow-up	[69,70,72]
**Treatment utility**	Phenotype predicts differential benefit in distress, functioning, eating behavior, or diet quality beyond EE score change	Stratified RCTs across weight groups with prespecified EE definitions and patient-relevant outcomes	[69]

BMI = body mass index; BED = binge eating disorder; BN = bulimia nervosa; EMA = ecological momentary assessment; RCT = randomized clinical trials.

Night eating syndrome offers a useful benchmark for this validation process: it is an eating-related residual-category construct that has undergone a formal, consensus-based diagnostic process, specifying frequency, awareness, and associated distress thresholds [64,65]. EE would require a comparably reproducible standard before any of the domains in Table 3 could be considered met. For nutritional practice, the useful question is not simply whether a patient reports EE but rather what nutritional manifestation follows the emotional trigger(s). Assessment can distinguish changes in food choice, diet quality, meal pattern, or preference for highly palatable and highly processed foods from episodes that are characterized primarily by loss of control or increased quantity [4]. This allows dietetic and psychological formulations to be linked without the assumption that all EE produce overeating or obesity. However, training coping mechanisms for negative emotions and nutrition education have been linked to a lower risk of EE in patients with obesity or overweight, especially in the presence of comorbid depression, anxiety, and stressful stimuli [73].

More recent evidence has strengthened this treatment-target perspective. A 2023 systematic review and meta-analysis of 34 studies revealed that EE interventions produced a small but significant reduction in weight (−1.08%, 95% CI [−1.66, −0.49]) and a greater reduction in EE scores (−2.37%, 95% CI [−3.76, −0.99]), with cognitive-behavioral approaches positioned among the more promising strategies; therefore, in adults with overweight or obesity, exploration of an underlying EE and targeting it could have beneficial outcomes [30]. The 2023 synthesis included 34 studies with differing designs and interventions, rather than 34 randomized trials, and its findings should be interpreted in the context of heterogeneity in samples, measures, comparators, and intervention delivery [30]. A 2025 synthesis (*N* = 6729 participants, n = 47 studies) highlighted self-regulation, psychological flexibility, self-compassion, and future-oriented strategies as promising behavior-changing techniques, concluding that weight management services should consider screening patients for EE in order to formulate more adequate therapeutic interventions [31]. In 2026, reductions in EE through Mindfulness-based Eating Awareness Training (*N* = 46 participants, 10-week online-delivered program) were reported among university students, and reductions through the Mind-Eat program (*N* = 66 adults, 12-week program) were reported among adults with overweight or obesity [74,75]. These two programs also showed reductions in negative psychosocial effects, helping individuals to cope better with stress through mindful eating and emotion regulation [74,75].

The intervention evidence summarized here is derived largely from adults with overweight or obesity, often in weight-management settings [30,31,75]. Although research in university students extends this literature [74], evidence of effectiveness specifically in adequately characterized normal-weight EE populations remains limited. Benefits observed in weight-management samples cannot be assumed to transfer unchanged to individuals of normal weight. Future trials would have to recruit across weight groups, report baseline EE definitions and clinical significance, and examine whether effects on distress, functioning, and eating behavior differ by weight status.

The field of EE therapy requires similar caution as other areas of nutrition-related neuroscience, e.g., although biologically plausible pathways and promising preclinical or preliminary clinical findings may justify further investigation, they do not, in and of themselves, establish clinical efficacy or support definitive recommendations for specific interventions [76]. Pharmacological data should be interpreted cautiously because the available evidence remains indirect in relation to an EE-specific indication. The 2025 meta-analysis of glucagon-like peptide-1 receptor agonists reported an improvement in restrained eating (g = 0.35, 95% CI [0.13, 0.57]) [77]. This research also mentioned a small improvement in emotional eating scores with GLP-1 receptor agonists versus placebo (Hedges g = 0.32, 95% CI [0.11,0.54]), based on three studies with 341 participants [77]. These secondary-outcome data from populations with overweight/obesity and/or diabetes are indirect in relation to an EE phenotype, and they do not support prescribing GLP-1 receptor agonists specifically for EE or demonstrate benefit independent of weight loss or other metabolic changes. Dedicated trials should prespecify EE outcomes, assess clinical impact, and test whether baseline phenotype predicts benefit beyond changes in body weight.

Beyond CBT and mindfulness-based approaches, the broader landscape of psychological and pharmacological treatments remains only indirectly informative for EE specifically. Enhanced CBT (CBT-E) includes a dedicated “mood intolerance” module for difficulties in coping with negative affect that trigger binge eating, procedurally overlapping with the emotion-regulation and distress-tolerance skills of dialectical behavior therapy [78]. However, a systematic review of third-wave behavioral therapies (dialectical behavior therapy, acceptance and commitment therapy, mindfulness-based interventions, schema therapy, and compassion-focused therapy) in eating-disorder samples found large pre-post symptom improvements but no consistent superiority over CBT or active comparators in randomized trials, so CBT retains its status as the recommended treatment for bulimia nervosa and binge-eating disorder, with interpersonal psychotherapy considered a strong alternative [79]. Pharmacological evidence outside the GLP-1 RA literature is indirect in relation to EE, so that data can only be extrapolated with a limited level of trust. For example, in a randomized trial of adults with moderate-to-severe binge-eating disorder, lisdexamfetamine reduced binge-eating days at 50 and 70 mg/day but not at 30 mg/day, thus informing but not establishing a targeted pharmacotherapy for an EE phenotype [80].

Clinical assessment and intervention need to consider emotional distress, functional impairment, diet quality, meal patterns, perceived control, and the patient’s own priorities alongside EE questionnaire scores. Treatment goals are required to focus on reducing distress and adverse consequences and improving flexible eating and daily functioning, since a lower EE score alone is not sufficient evidence of meaningful benefit. Emotion-related eating that is adaptive, culturally congruent, and unaccompanied by impairment should not automatically become a treatment target [69,71].

## 9. Limitations and Research Priorities

This article is intentionally structured as a conceptual analysis rather than a systematic or scoping review; therefore, the possibility of missing relevant data through a non-systematic search is to be considered possible. Selection bias cannot be excluded, and the absence of retained search strings limits the reproducibility of the literature search. The construct of EE was defined in foundational studies, with particular emphasis on developments from recent years that change how EE can be phenotyped, measured, and clinically interpreted. The aim was not an exhaustive capture of the literature but rather a testable position on clinical and nutritional validation. Heterogeneity in EE definitions, populations, instruments, and study designs remains a major limitation, and this is why the proposed framework is hypothesis-generating and requires prospective validation.

The interpretation is also constrained by possible publication and selective-reporting biases, which were not formally assessed, due to the non-systematic approach of this article. Much of the literature discussed relies on self-report and cross-sectional designs, leaving findings vulnerable to recall and attribution biases, shared-method variance, reverse directionality, and residual confounding. Also, it is to be noted that differences in EE definitions, emotional valence, instruments, recall periods, and thresholds limit comparability. Some studies include only or predominantly women, and reported sex or gender differences cannot necessarily be separated from recruitment, measurement, and contextual influences. Cultural differences are also important to assess in relation to emotional expression, food practices, dietary restraint, and the meaning of distress, which may further limit transferability. These considerations preclude assuming that the proposed dimensions or future thresholds will operate equivalently across populations without direct testing. The concentration of intervention evidence in populations with overweight or obesity further limits generalizability to individuals of normal weight.

The first priority identified by this article is longitudinal phenotyping in EE. Large, adequately powered longitudinal cohorts with extended follow-up and repeated EMA assessment periods are needed to distinguish relatively stable between-person EE tendencies from within-person, state-dependent fluctuations. Studies should examine persistence, recurrence, and change across contexts and, if reproducible profiles emerge, transitions between them without assuming a fixed sequence. Candidate quantitative boundaries for frequency, persistence, control, distress, and impairment should be evaluated against clinical and functional outcomes and validated in independent samples rather than imposed by the conceptual framework. Prediction should be tested beyond relevant established factors, including mood and anxiety symptoms, binge eating, restraint, sleep, and BMI [69,70]. Sampling across weight groups, developmental stages, sex and gender, and cultural settings is necessary to evaluate generalizability. Developmental studies are particularly important because emotional regulation, body image, and weight status interact differently across childhood, adolescence, and adulthood [23,24,25,41].

Second, measurement should be explicitly multimethod. Future research should prespecify whether its primary construct is the perceived tendency to eat in response to emotion, the within-person temporal association between affect and subsequent eating, or both as separate outcomes. Operational definitions need to identify emotional valence and intensity, the time window, whether intake increases or decreases, and whether quantity, food choice, timing, or control is assessed. N-EE and P-EE are to be reported and analyzed separately instead of being collapsed into a single undifferentiated estimate [2,3,28,36]. Questionnaires can be combined with repeated EMA and objective or semi-objective dietary measures where feasible, in order to examine how perceived emotional eating tendencies relate to within-person associations between emotional states and subsequent eating behavior. Temporal analyses are required to account for relevant contextual influences, including hunger, dietary restraint, sleep, and social setting, and affect preceding eating should not automatically be interpreted as proof of causation.

Third, future studies should be conducted to test clinical utility rather than only assessing associations. Clinical utility requires evidence that identifying a candidate EE phenotype improves management or prognosis, alongside evidence for its validity. Stratified trials could ask whether patients with high negative EE benefit preferentially from emotion-regulation or mindfulness-focused interventions, as restraint-dominant or reward-dominant phenotypes require different approaches. Such an approach is expected to favor the transition of EE from a purely descriptive label into a precision-treatment variable.

Fourth, nutritional phenotyping should accompany psychological phenotyping. Future cohorts and intervention trials should determine whether EE subtypes show reproducible differences in diet quality, food choice, degree of processing, meal timing or regularity, waist-related outcomes, and metabolic risk beyond that of BMI alone [4,27]. This could test whether nutritional information adds incremental clinical value rather than merely redescribing obesity.

## 10. Conclusions

The clinical and nutritional relevance and potential modifiability of EE have been supported by the cumulative literature, which has demonstrated substantial variation across populations, emotional contexts, dietary manifestations, and assessment methods. Retrospective questionnaire scores, associations with obesity, and treatment responsiveness alone cannot be used to establish EE as a distinct disorder.

Within the limits of this conceptual analysis, a dimensional, transdiagnostic nutritional-behavioral framework appears useful for organizing the heterogeneity of EE and guiding research. This proposal does not establish the superiority of one classification model or exclude future categorical recognition, since such recognition would require reproducible evidence of clinical significance, diagnostic distinctiveness, temporal course, and added clinical utility. Loss of control, persistence, nutritional correlates, and prognosis remain important dimensions to investigate and are not established diagnostic criteria.

## Figures and Tables

**Table 1:** **Arguments for and against independent diagnostic recognition of emotional eating.**

Domain	Evidence Supporting Clinical Relevance	Current Limitation
**Phenomenology**	Recurrent emotion-linked eating is clinically recognizable and associated with distress in some individuals	Emotion-related eating also occurs normally; positive and negative EE differ
**Psychometrics**	Multiple established instruments quantify EE and related eating styles	Scales, EMA, and laboratory measures may assess related but distinct constructs; incomplete convergence does not by itself invalidate a method
**Cross-diagnostic relevance**	EE is elevated in cases of obesity and several ED phenotypes	There is a strong overlap with BED, BN, depression, anxiety, restraint, and body-image concerns
**Course and prognosis**	Prospective links with depressive symptoms and later weight-related outcomes have been reported	Long-term stability and incremental prognosis of a distinct EE syndrome remain insufficiently demonstrated
**Treatment**	CBT, mindfulness, self-regulation, and broader behavioral interventions can reduce EE scores, mainly in samples with overweight or obesity	Response is not specific to a unique syndrome; lower scores alone do not establish meaningful clinical benefit
**Nutritional/biological relevance**	Dietary-pattern associations and secondary eating-behavior outcomes in GLP-1 receptor agonist trials provide indirect evidence relevant to nutritional and biological pathways	Secondary eating-behavior outcomes do not establish EE-specific efficacy or diagnostic independence.

BED = binge eating disorder; BN= bulimia nervosa; CBT = cognitive-behavioral therapy; ED = eating disorder; EE = emotional eating; EMA = ecological momentary assessment; GLP-1 = glucagon-like peptide 1.

**Table 2:** **Differential positioning of emotional eating among related constructs.**

Construct	Core Feature	Relationship to Emotion	Key Distinction from EE
**Emotional eating [1,11]**	Changes in intake quantity, food choice, meal timing or patterns, and perceived control associated with emotional states	Central by definition	May occur with or without loss of control, objective binge size, obesity, clinically significant distress, or a formal eating disorder
**Binge-eating disorder [51,52]**	Recurrent objectively large binge episodes with loss of control	Negative affect may trigger episodes	Diagnostic criteria include binge frequency, duration, distress, and associated features
**Bulimia nervosa [51,52]**	Recurrent binge eating with compensatory behaviors (self-induced vomiting, laxative misuse, excessive exercise) and overvaluation of body shape/weight	Negative affect frequently precedes and follows binge-purge episodes	Requires compensatory behavior and body shape/weight overvaluation; EE carries neither a compensatory nor a cognitive-shape criterion
**Restrained eating [14,60]**	Cognitive effort to restrict intake	Emotion may disrupt restraint	Restriction is primary; overeating is not needed
**External eating [17]**	Eating in response to food-related external cues	Emotion is not necessary	The defining trigger is cue reactivity rather than affect
**Mood-related appetite change [51,52,61]**	Appetite/intake change occurring within a mood syndrome	Linked to mood state	Typically conceptualized as a symptom of a mood disorder unless an independent eating pattern is established
**Sleep-related eating disorder [58]**	Involuntary or partially conscious eating during the main sleep period	Emotion is not the defining trigger	Altered awareness and sleep-state context distinguish it from conscious EE
**Loss-of-control eating [62]**	A subjective sense of being unable to stop eating, independent of the objective amount consumed	Often emotion-triggered, in association with negative affect	Defined by the control dimension itself, not the emotional antecedent; can occur without a discernible emotional trigger or objectively large intake
**Food addiction [63]**	Addictive-like eating assessed via substance-use-derived criteria (loss of control, tolerance, withdrawal-like symptoms, continued use despite consequences), usually toward hyperpalatable foods	Negative affect often cited as a craving/relapse trigger, paralleling substance-use models	Organized around a substance-use-disorder framework rather than the emotional state itself; overlaps with N-EE but imports addiction-model assumptions that EE does not require
**Night eating syndrome [64,65]**	Recurrent evening hyperphagia and/or nocturnal eating, with awareness and recall, accompanied by distress or impairment	Emotional distress may accompany episodes but is not the defining trigger	Primarily defined by the timing of eating relative to the sleep–wake cycle; on the contrary, EE is defined by its relationship to emotional states and may occur at any time

EE = emotional eating; N-EE = negative-emotional eating.

## Data Availability

No new datasets were generated or analyzed for this article.

## Abbreviations

The following abbreviations are used in this manuscript:

Abbreviation	Definition
BED	Binge-eating disorder
BMI	Body mass index
BN	Bulimia nervosa
CBT	Cognitive behavioral therapy
CBT-E	Enhanced cognitive behavioral therapy
CI	Confidence interval
COVID-19	Coronavirus disease 2019
DEBQ	Dutch Eating Behavior Questionnaire
DSM-5-TR	Diagnostic and Statistical Manual of Mental Disorders, Fifth Edition, Text Revision
ED	Eating disorder
EE	Emotional eating
EES	Emotional Eating Scale
EMA	Ecological momentary assessment
GLP-1	Glucagon-like peptide-1
GLP-1 RA	Glucagon-like peptide-1 receptor agonist
HPA	Hypothalamic–pituitary–adrenal
ICD-11	International Classification of Diseases, 11th Revision
MEDLINE	Medical Literature Analysis and Retrieval System Online
N-EE	Negative emotional eating
OSFED	Other specified feeding or eating disorder
P-EE	Positive emotional eating
PNEES	Positive–Negative Emotional Eating Scale
RCT	Randomized controlled trial
SEES	Salzburg Emotional Eating Scale
SMD	Standardized mean difference
TFEQ	Three-Factor Eating Questionnaire
TFEQ-R18	Revised 18-item Three-Factor Eating Questionnaire
UFED	Unspecified feeding or eating disorder
